# Data-guide for brain deformation in surgery: comparison of linear and nonlinear models

**DOI:** 10.1186/1475-925X-9-51

**Published:** 2010-09-15

**Authors:** Hajar Hamidian, Hamid Soltanian-Zadeh, Reza Faraji-Dana, Masoumeh Gity

**Affiliations:** 1 Control and Intelligent Processing Center of Excellence (CIPCE), School of Electrical and Computer Engineering, University of Tehran, Tehran, Iran; 2 Radiology Image Analysis Lab., Henry Ford Hospital, Detroit, MI 48202, USA; 3 School of Electrical and Computer Engineering, University of Tehran, Tehran, Iran; 4 Department of Radiology, Tehran University of Medical Sciences, Tehran, Iran

## Abstract

**Background:**

Pre-operative imaging devices generate high-resolution images but intra-operative imaging devices generate low-resolution images. To use high-resolution pre-operative images during surgery, they must be deformed to reflect intra-operative geometry of brain.

**Methods:**

We employ biomechanical models, guided by low resolution intra-operative images, to determine location of normal and abnormal regions of brain after craniotomy. We also employ finite element methods to discretize and solve the related differential equations. In the process, pre- and intra-operative images are utilized and corresponding points are determined and used to optimize parameters of the models. This paper develops a nonlinear model and compares it with linear models while our previous work developed and compared linear models (mechanical and elastic).

**Results:**

Nonlinear model is evaluated and compared with linear models using simulated and real data. Partial validation using intra-operative images indicates that the proposed models reduce the localization error caused by brain deformation after craniotomy.

**Conclusions:**

The proposed nonlinear model generates more accurate results than the linear models. When guided by limited intra-operative surface data, it predicts deformation of entire brain. Its execution time is however considerably more than those of linear models.

## Background

Medical imaging methods play a key role in localizing tissues and organs during surgery. Pre-operative imaging devices generate high-resolution images of the tissues and organs while intra-operative imaging devices generate their low-resolution images. The pre-operative images however cannot be easily used during surgery since they do not reflect correct anatomy and geometry of tissues and organ intra-operatively. This is due to motions and deformations of soft tissues over time. The end result is that actual positions of the tissues during surgery do not match with those reflected in their preoperative images. To be able to use pre-operative images intra-operatively, they should be deformed based on the tissue geometry reflected in the intra-operative images. However, intra-operative images are low resolution and low quality. To overcome these limitations, intra-operative images are used along with biomechanical models to update pre-operative images such that they reflect the tissue geometry during surgery [1-6]. In this process, the Finite Element Method (FEM) [7] is employed to solve the partial differential equations that govern deformation behavior of soft tissues.

In our previous study [8], we used the finite element method to develop and compare two linear models: mechanical and elastic [9-12] for image-guided neurosurgery. We showed that accurate computation of brain deformation due to craniotomy can be achieved by defining a load through prescribed displacements of the corresponding points in the pre- and intra-operative images. Experimental results showed that the mechanical model was superior to the elastic model; the brain deformation could be estimated by the mechanical model more accurately. The execution time of the mechanical model was however about 50% more than that of the elastic model.

In this paper, a nonlinear model is developed for estimating the brain deformation and compared to the linear mechanical model. The mechanical model [13,14] is based on the principle that the sum of the virtual work from the internal strains is equal to the work from the external loads. In this formulation, the brain deformation is assumed to be infinitesimal, the brain tissue is treated as an elastic material, and the relation between strain and stress is linear. The nonlinear model [15], on the other hand, is based on the equation of equilibrium that relates the covariant differentiation of stress (with respect to the deformed configuration) to the body force per unit mass. In this model, the brain deformation may be large, brain tissue is treated as a hyper visco-elastic material, and the stress-strain behavior of the tissue is non-linear [16,17].

To solve the equations of the models, actual values of the organ parameters are needed. To this end, we optimize the initial, approximate values to obtain the actual values. The cost function for this optimization is the distance between the estimated positions of the pre-operative anatomical landmarks and their corresponding actual positions in the intra-operative images. One half of these landmarks are utilized in the optimization process and the other half in the evaluation process. We compare the models using their errors on simulated and real data sets, using the corresponding points that are not used in the optimization process.

In the next section, the proposed models, meshing, and boundary conditions are explained. Optimization of the parameters of the models is also described in this section. In Section 3, the results obtained for a test sphere as a model of the brain and real brain extracted from MRI are presented. Finally, Section 4 summarizes the conclusions of the work.

## Methods

### Construction of 3D Model and Finite Element Mesh

**Figure 1:**
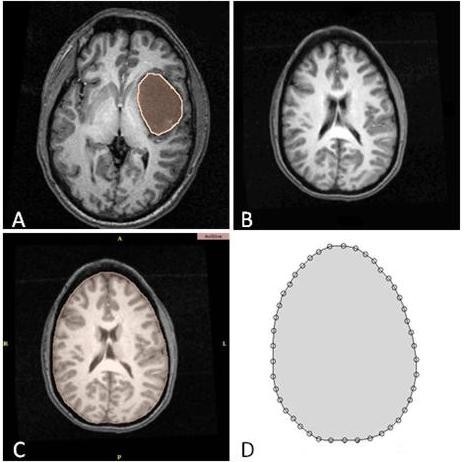
**Segmented MRI of the head used to construct a patient specific brain mesh**. (a) Tumor segmented in a pre-operative image. (b) Another pre-operative slice of the brain. (c) Brain segmented from image (b). (d) Contour of the brain. Pre-operative images are used to construct computational models of the brain and tumor while the intra-operative images are used to measure the displacements of the anatomical landmarks.

Patient-specific geometric data are obtained from a set of six pre-operative and intra- operative MRI of patients undergoing brain tumor surgery. The human studies were reviewed and approved by the IRB office of the Brigham and Women's Hospital (Harvard Medical School, Boston, Massachusetts, USA). The pre- and intra-operative images are registered rigidly in the Surgical Planning Laboratory. In order to distinguish between the brain parenchyma and tumor, the corresponding regions of the images are segmented manually using the 3D-Slicer software (open-source software for visualization, registration, segmentation and quantification of medical data; see http://www.slicer.org for details). The results are two-dimensional contours a sample of which is shown in Figure 1.

**Figure 2:**
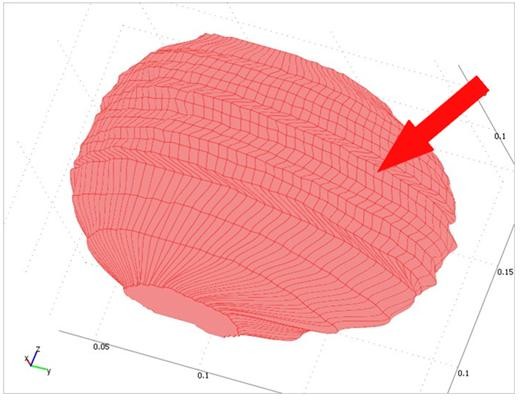
**For creating the 3D model, the slices that are near the craniotomy surfaces have higher resolutions than the slices far from the surface**. For the craniotomy surface, we choose more slices than the other parts because if all parts have the same number of slices, the implementation will be time consuming or the accuracy of the model will be insufficient if the total number of slices is small.

From the two-dimensional contours, the surface and volumetric patches are created using the COMSOL3.3 software (see http://www.comsol.com for details) to represent three-dimensional models of the surfaces and volume of the brain parenchyma and tumor. This software is based on the finite element methods developed for solving partial differential equations, visualization, and meshing and has strong post-processing modules. For creating the 3D model, the slices that are near the craniotomy surfaces have higher resolutions than the slices far from the surface. This means that for each case, we have a specific number of slices with a specific thickness. For the craniotomy surface, we choose more slices than the other parts because if all parts have the same number of slices, the implementation will be time consuming or the accuracy of the model will be insufficient if the total number of slices is small. Therefore, in the craniotomy part, the contours are selected very close to each other and in other parts they are selected relatively far from each other as shown in Figure 2. Also, for the tumor, we use close contours.

**Figure 3:**
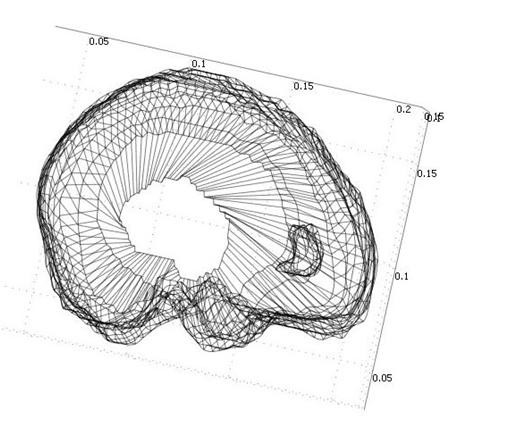
**Final model of parenchyma and tumor**. The models of parenchyma and tumor are created individually and then combined to create a model of the brain.

**Figure 4:**
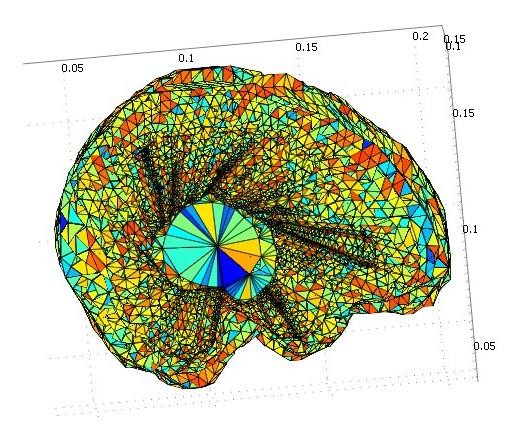
**Patient-specific brain mesh consisting of 41,617 tetrahedral elements**. After creating the 3D models, automatic 4-noded tetrahedral meshes with Lagrange shape functions are generated for both of the parenchyma and the tumor using the COMSOL3.3 software.

Figure 3 shows the final models of the tumor and the brain such that both volumes can be seen. After creating the 3D models, automatic 4-node tetrahedral meshes with Lagrange shape functions are generated for both of the parenchyma and the tumor using the COMSOL3.3 software. An example of the mesh generated by the software is shown in Figure 4, which consists of 41,617 tetrahedral elements.

### Biomechanical Models of Brain

The biomechanical models guided by low-resolution intra-operative images may be used for updating the high-resolution pre-operative images [18,19]. These models can be linear or nonlinear. In our previous work [8], we compared two linear models (elastic and mechanical) and found that the mechanical model generated more accurate results. In this paper, we develop a nonlinear model and compare it to the best of the two linear models studied previously, i.e., the mechanical model. Relying on the study of [13], the initial coefficients of the mechanical model are set to Young modules = 3 kPa and Poisson ratio = 0.45. Next, we explain the nonlinear model. The readers are referred to our previous work [8] for the details of the mechanical model.

### Non-linear Model

In this model, the brain is supposed to be a single-phase continuum undergoing large deformations. In this analysis, the stresses and strains are measured with respect to the current configuration. Therefore, using Almansi strain and Cauchy stress [20], the virtual work principle can be written as:

(1) $$\int_{V}\tau_{ij}\delta \varepsilon_{ij}dV=\int_{V}f_{i}^{B}u_{i}dV+\int_{S}f_{i}^{S}u_{i}dS,$$

where $\int_{V}\tau_{ij}\delta \varepsilon_{ij}dV$ is the internal virtual work of the strain, $\int_{V}f_{i}^{B}u_{i}dV$ is the virtual work of the external force applied to the entire body, $\int_{S}f_{i}^{S}u_{i}dS$ is the virtual work of the external force applied to the surface, and *u *is the displacement parameter. As the brain deforms, the volume *V *and surface *S *in the integrals of equation (1) change. As such, they are part of the solution and the input data can be used to set their initial values. To find their values after deformation, appropriate finite element procedures can be used to solve equation (1) with equation formula that describes the mechanical property of the material, i.e., appropriate constitutive models. To this end, we use the nonlinear model proposed by [21,22]. This model is suitable for the low strain rates that are typical in surgical procedures and is described in equation (2).

(2) $$\begin{array}{l} W=\int_{0}^{t}\{\sum_{i+j=1}^{N}\left[C_{ij0}\left(1-\sum_{k=1}^{n}g_{k}(1-e^{-(t-\tau )/\tau_{k}})\right)\right]\\ \times \frac{d}{d\tau }[{\left(J_{1}-3\right)}^{i}{\left(J_{2}-3\right)}^{j}]\}d\tau \end{array}$$

where *τ_k _*are characteristic times, *g_k _*are the relaxation coefficients, *N *is the order of polynomial in strain invariants, and *J_1_, J_2 _*are strain invariants as described by:

(3) $$\begin{array}{l} J_{1}=Trace[B],\;\\ J_{2}=\frac{J_{1}^{2}-Trace[B^{2}]}{2J_{3}},\\ J_{3}=\sqrt{\det B} \end{array}$$

where *B *is the left Cauchy*-*Green strain tensor. We use the stationary form of the equation (2) because we solve the problem for the steady state form of deformation when the deformation of the brain is stabilized. All details about this equation, how their parameters are determined, and how the stationary form of the equation can be found, are described in [22]. The initial values of the model's parameters are taken from [22] for n = 2, N = 2 as summarized in Table 1.

**Table 1:** Parameters used for the nonlinear model as the initial values for the optimization process.

Instantaneous Response	Characteristic Time	Characteristic Time
C100 = 263 (Pa)	τ_1 _= 0.5 (s)	τ_2 _= 50 (s)
C010 = 263 (Pa)		
	
C110 = 0 (Pa)	**Instantaneous Elasticity**	**Instantaneous Elasticity**
	
C020 = 491 (Pa)	g_1 _= 0.450	g_2 _= 0.365
C200 = 491 (Pa)		

### Boundary Conditions

**Figure 5:**
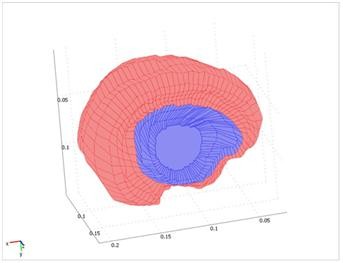
**Boundary condition for the displacement parameter**. In both models, the exposed surface is assumed free to move and the remaining surfaces are fixed. Therefore, the parameter *u *is estimated for the exposed surfaces (blue surfaces) and the surfaces close to it but it is zero for the remaining surfaces (red surfaces).

To solve the equations governing the models, boundary conditions should be specified. To this end, we assume that the exposed surface and the nearby regions are free to move but the remaining surfaces are fixed. The exposed surface is defined manually using the intra-operative images. Thus, the parameter *u *is free to vary on the exposed surfaces and the surfaces near it, and is zero on the remaining surfaces. However, it is unconstrained for the inside of the volume. Figure 5 shows the boundary condition for a sample case. The exposed surface is shown in blue and the fixed surface is shown in red. Note that when the skull is exposed, the exposed and the nearby tissues deform.

In both models, we have conditions for the force (*F*) variable rather than the displacement variable. Previous works suggest that this parameter is a constant (fixed) value for each surface and determine its value for each surface by registering the intra- and pre-operative volumes [13]. We fix this parameter for the center of the exposed surface and let it change for the remaining exposed surface. We also use *F = u *for the boundary conditions of the fixed boundary nodes because all of the equations lead to the equation *Ku = F *(*K *encapsulates all coefficients of the equation) and therefore, according to [13], the non-diagonal elements of the rigidity matrix *K *for which the deformation is supposed to be known are zero and the diagonal elements are one. Further details can be found in [13].

**Figure 6:**
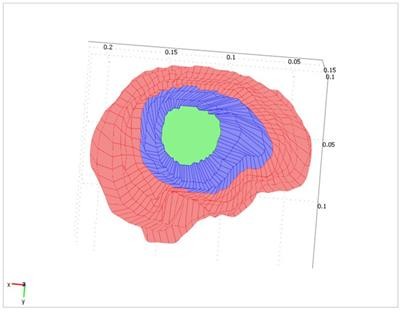
**For both models, there are conditions for the force variable (*F*)**. The value of *F *for the green surface (the center of the exposed surface) is fixed, for the blue surface (the remaining exposed surface) is unconstrained, and for the red surface (unexposed surface) is zero.

In other words, no forces are applied to the fixed surface so the equivalent force for this surface will be zero. The initial value of the parameter *F *for the center of the exposed surface is set by examining the MR images of six patients. The exact value of *F *for each part is determined by the optimization process as explained in the next section. This condition is illustrated in Figure 6. The value of parameter *F *for the green surface (the center of the exposed surface) is fixed, for the blue surface (the remaining exposed surface) is unconstrained, and for the red surface (unexposed surface) is zero.

### Optimization Process

The parameters of the models change from case to case. Thus, as in our previous work [8], we use approximated parameters as the initial values and optimize them for each case to maximize the accuracy of the results for the known deformations of each case. As mentioned before, we propose a new approach for determining the parameter *F*. The value of this parameter in the center of the exposed surface is also determined in the optimization process. To this end, we choose a cost function defined as the sum of the distances between the actual positions of the anatomical landmarks in the intra-operative images and their corresponding estimated positions based on the deformation results of applying the two models on the pre-operative images.

**Figure 7:**
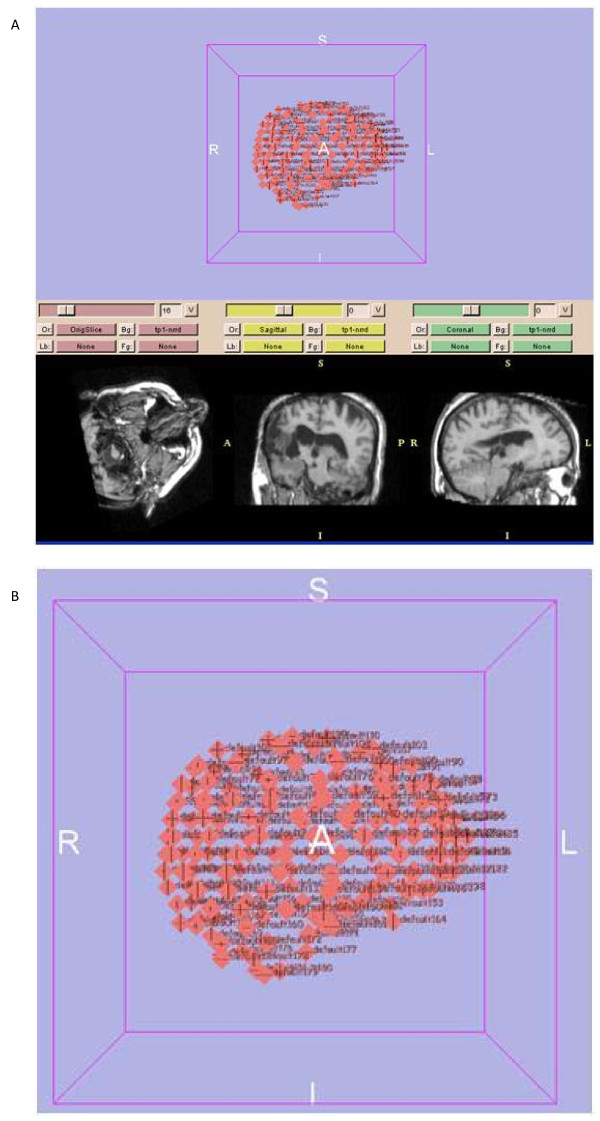
**Illustration of using the 3D-Slicer software for manual selection of the landmarks by expert radiologist**. We select points from all 2 D images of the brain. a) The software saves and shows the 3D coordinates of the selected points. b) A zoomed version of the 3D image is shown for better illustration.

Displacements of the landmarks are determined by an expert physician who uses the 3D-Slicer software to mark the corresponding pre- and intra-operative points. The models are then applied to the pre-operative points and their results compared with the corresponding actual results. Figure 7 shows the screen of the software used to define the points. When the expert selects points on the 2 D images of the brain, the coordinates of the point in the 3D space are shown and saved in a text file. The points are mostly selected near the exposed surface due to larger displacements of the points in this area relative to the other points. Note that the size of the exposed area and the consequent deformation of the brain are not the same in all cases. For instance, if the tumor is large, the surgeon opens a relatively large surface of the brain and therefore, the deformations are large. In this case, the expert selects several points for accurate estimation of the parameters. In total, our expert has selected about 70 pairs of the corresponding landmarks for each case. One half of the landmarks have been used in the optimization process and the other half in the testing process. We use the Maltlab optimization toolbox (fminsearch function) to optimize the cost function and find the optimal values of the parameters as explained next.

In both models, we do not know the exact value of the force applied to the center of the exposed surface of the brain. The value of this parameter determined in sample cases is used as an initial value and the optimal value is determined by the proposed optimization process. In addition, in the mechanical model, the two parameters (Young modulus and Poisson's ratio) reported in the literature are not the same for different patients and thus they are also optimized for each case. For the nonlinear model, in addition to the initial value for F, the parameters listed in Table 1 are used in the optimization process except the parameters of characteristic time. This is because we study the problem in the steady state which is independent of these parameters. Also, the parameters g_1 _and g_2 _lead to g_1 _+ g_2 _= g in the equation of the steady state. Therefore, these values are varied to find the minimum error.

## Results

### Simulation Data

**Figure 8:**
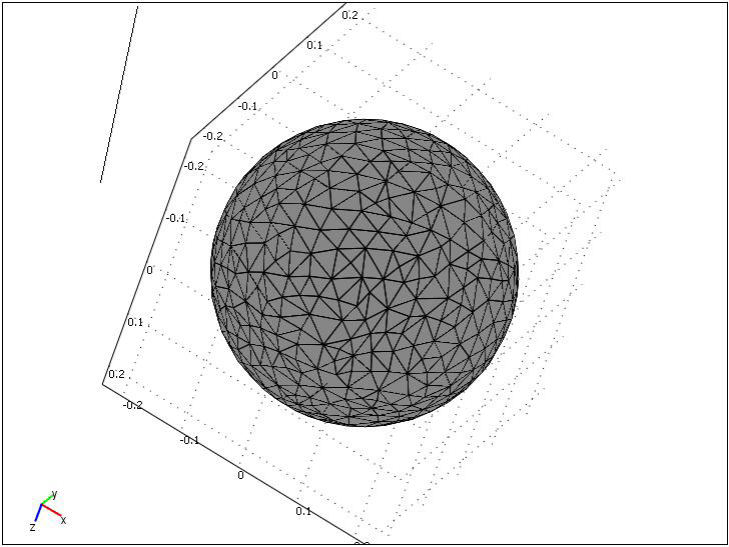
**Sphere mesh consisting of 9,028 tetrahedral elements**. After creating the 3D models, automatic 4-noded tetrahedral meshes with Lagrange shape functions are generated for the sphere using the COMSOL3.3 software.

For evaluation of our method, we first apply the models on a brain simulation (a sphere with the diameter of 22 Cm). To model the skull opening, we assume that one section of this sphere is exposed and the other sections are fixed. In the meshing process, we use 9,028 tetrahedral meshes. Figure 8 shows an example of meshing for the spherical brain model. For each model, we use the brain model with specific parameters and boundary conditions.

We specify a set of anatomical landmarks for the optimization process and another set for the evaluation of the optimization results. After the optimization, a comparison of the cost function for the evaluation landmarks shows whether the brain deformation is reliably modeled and if the optimization process estimates the model parameters accurately. In this study, we have used 10 points of the sphere for the optimization process and another 10 points for the evaluation of the results. To implement the models, we have used the COMSOL3.3 software which is based on the finite element methods for solving partial differential equations.

**Figure 9:**
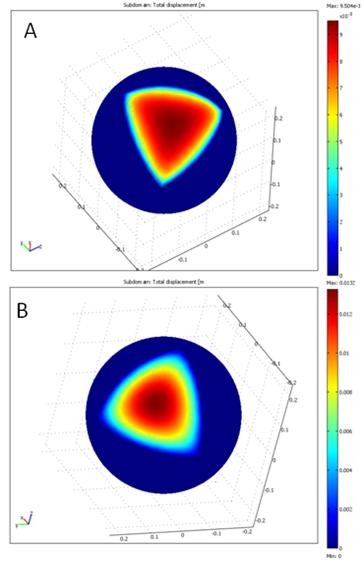
**Deformation results of a sphere as a simple model of the brain**. a) Using the linear mechanical model and b) Using the nonlinear model. Note that the deformations predicted by the linear mechanical model and nonlinear model are smooth, similar to the brain deformations.

Figure 9 shows the results of the two models for the sphere. Note that deformations of the models are smooth and realistic, similar to those of the brain. This is because the models solve their equations assuming that the equivalent work applied to a surface is zero. Although the results are similar but as we will see next, the results of the nonlinear model are more desirable than those of the other model. For the mechanical and nonlinear models, the mean errors of the points used in the optimization process are 0.1172 mm and 0.0683 mm, respectively. The mean errors of the points not used in the optimization process are 0.2731 mm and 0.1816 mm, respectively. Therefore, accuracy of the nonlinear model is higher than that of the mechanical model. This is because the nonlinear model is more flexible than the linear model. In addition, the number of the parameters of the nonlinear model is larger than that of the mechanical model. Consequently, the nonlinear model fits the landmark data more closely than the mechanical model.

Table 2 shows the assumed and estimated parameters of the two models. As seen, the optimization process estimates the parameters of the nonlinear model more closely than those of the mechanical model. Comparing the final values of the cost function for the points used in the optimization process and those not used in the optimization process, we conclude that the nonlinear model is more appropriate than the linear, mechanical model. However, the execution time of the nonlinear model is six times of the mechanical model. The mean execution time is approximately 35 hours for the linear model and 199 hours for the nonlinear model for each case. Both models are implemented on a PC with the 1.86 GHz CPU and 4 GB RAM.

**Table 2:** Assumed and estimated parameters and their variations for the models using a sphere.

Mechanical model	Young modulus		Poisson's ratio		Force	
**Assumed**	0.45		3000		Fx = 1500	
					Fy = 1500	
					Fz = 1500	

**Estimated**	0.45 ± 0.0056		3000 ± 175.6		Fx = 1500 ± 90.8	
					Fy = 1500 ± 87.9	
					Fz = 1500 ± 93.2	

**Nonlinear model**	**C100**	**C010**	**C200**	**C020**	**g_1_+g_2_**	**Force**

**Assumed**	263	263	491	491	0.815	Fx = -300
						Fy = 300
						Fz = 300

**Estimated**	263 ±4.0401	263 ±7.8404	491 ±14.4139	491 ±13.4578	0.815 ±0.0083	Fx = -300 ± 9.1726
						Fy = 300 ± 10.0278
						Fz = 300 ± 8.7998

We use sphere as a simple model of the brain. Specific parameters are assumed and the resulting deformations of specific points are used in the optimization process. Other points are used for testing of the optimization process. Then, the parameters are changed and the optimization process is repeated to estimate the model parameters again. The results illustrate that the brain deformation can be modeled by the proposed models and the proposed optimization method can accurately estimate the model parameters. For each model, we have done this process and have compared the accuracy of models using the matching errors of the points used in the optimization process and those that are not used. Note that the optimization process estimates the parameters of the nonlinear model more accurately (with less bias and variance) compared with the mechanical model.

### Real Data

To evaluate the methods on the real data, we have used six image sets each containing 90 slices with 2.5 mm thickness and 286x286 pixels with 0.86 mm pixel size. Each image set contains both of the pre-operative and intra-operative MRI studies of a brain tumor patient who has undergone surgery.

It is commonly acknowledged that tumors are associated with ''stiffer'' tissue relative to the normal tissues. However, the volume of a tumor is usually small relative to the volume of the brain. Thus, uncertainties about the tumor's mechanical properties do not significantly affect the overall displacement field. Consequently, the tumor was simulated using the same constitutive model as ''healthy'' brain tissue. Also, the parameters of the two models for the tumor are equal to the brain's parameters [20]. Of course, if specific model parameters are known for the tumor, they can be used in the proposed algorithm.

**Figure 10:**
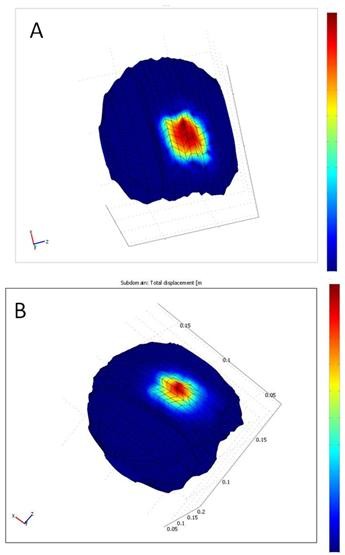
**3D results of optimizing the parameters of the linear mechanical and nonlinear models for the real brain data**. Note that the maximum deformation is shown in red and the minimum deformation is shown in blue. a) The result of linear mechanical model. b) The result of nonlinear model. As seen, the brain deformation in both models is smooth.

Sample results for the mechanical and nonlinear models are presented in Figure 10. The figure shows 3D results for a representative brain where large deformation is shown in red and small deformation is shown in blue. Note that the brain deformations in the two models are smooth. The results indicate that our simulations are realistic.

**Figure 11:**
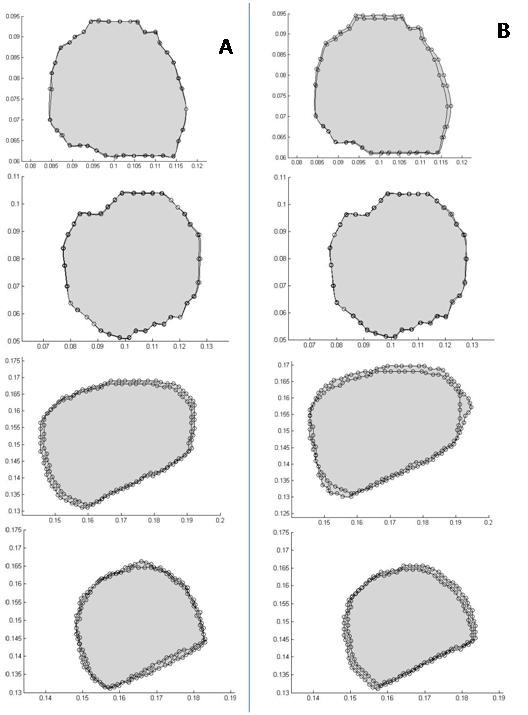
**Comparing the 2 D contours of the tumor in the coronal sections obtained from the intra-operative images with the results of the optimization process**. Column (a), the results of the nonlinear model, and column (b), the results of the linear mechanical model. As seen, the predicted results of the nonlinear model show a higher level of matching. It must be noted that these results show the tumor slices close to the craniotomy surface of the skull.

Figures 11(a)-(b) compare the 2 D contours of the tumor in the coronal sections obtained from the intra-operative images with the results of the optimization process for the two models. Note that the predicted results of nonlinear model show higher levels of matching than those of the linear model. These results show the tumor slices near the craniotomy surface. For the slices deep in the brain or the slices far from the craniotomy, the results of the two models show similar matching; both models follow the deformation quite well. This is because deformations of the brain tissues far from the craniotomy are small.

**Figure 12:**
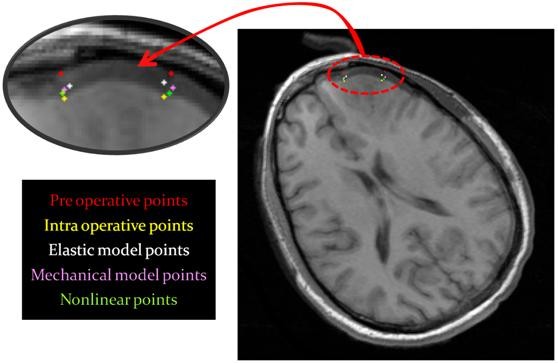
**Two groups of resulting points of each model and the corresponding real result from the intra-operative images**. The points estimated by the nonlinear model (green points) are the nearest points to the intra-operative points (yellow points) while those estimated by the linear mechanical model (pink points) are superior to those estimated by the linear elastic model.

Figure 12 shows the landmark locations estimated by the models and the corresponding actual results from the intra-operative images. Note that the points estimated by the nonlinear model (green points) are the closest to the intra-operative points (yellow points). Also, the points estimated by the linear mechanical model (pink points) are closer to the real results than those estimated by the linear elastic model (the elastic model is described in [8]. The numerical values of the maximum and mean errors of the testing landmarks in six cases are presented in Table 3. Table 4 presents the variations of the estimated parameters of the linear and nonlinear models in six cases relative to the initial values. The testing landmarks are mostly near the exposed surface. Again, the nonlinear model shows superior performance compared with the linear mechanical model. The errors depend on how much the brain surface is exposed, how much the CSF drains, and in general how much the brain conditions change due to the craniotomy. In addition, the position, the depth, and the size of the tumor affect the results. Finally, selection of landmarks has an important effect on the results; for a conservative evaluation, critical landmarks with relatively large displacements after opening the skull should be considered.

**Table 3:** Maximum and mean errors for the linear mechanical and nonlinear models.

	Case 1	Case 2	Case 3	Case 4	Case 5	Case 6
**Max error of the mechanical model**	$$\begin{array}{l} \Delta x=3.0mm\\ \Delta y=3.3mm\\ \Delta z=\text{1}\text{.1}mm \end{array}$$	$$\begin{array}{l} \Delta x=3.3mm\\ \Delta y=3.2mm\\ \Delta z=1.0mm \end{array}$$	$$\begin{array}{l} \Delta x=3.4mm\\ \Delta y=4.4mm\\ \Delta z=\text{0}\text{.3}mm \end{array}$$	$$\begin{array}{l} \Delta x=2.9mm\\ \Delta y=3.1mm\\ \Delta z=\text{0}\text{.8}mm \end{array}$$	$$\begin{array}{l} \Delta x=2.7mm\\ \Delta y=2.8mm\\ \Delta z=\text{0}\text{.4}mm \end{array}$$	$$\begin{array}{l} \Delta x=4.0mm\\ \Delta y=3.4mm\\ \Delta z=1.7mm \end{array}$$

**Mean error of the mechanical model**	$$\begin{array}{l} \Delta x=1.2mm\\ \Delta y=1.2mm\\ \Delta z=\text{0}\text{.4}mm \end{array}$$	$$\begin{array}{l} \Delta x=1.3mm\\ \Delta y=1.0mm\\ \Delta z=\text{0}\text{.3}mm \end{array}$$	$$\begin{array}{l} \Delta x=1.3mm\\ \Delta y=1.7mm\\ \Delta z=\text{0}\text{.1}mm \end{array}$$	$$\begin{array}{l} \Delta x=0.9mm\\ \Delta y=1.\text{1 }mm\\ \Delta z=\text{0}\text{.6 }mm \end{array}$$	$$\begin{array}{l} \Delta x=0.9mm\\ \Delta y=\text{0}\text{.8}mm\\ \Delta z=\text{0}\text{.1 }mm \end{array}$$	$$\begin{array}{l} \Delta x=1.0mm\\ \Delta y=0.7mm\\ \Delta z=\text{0}\text{.1}mm \end{array}$$

**Max error of the nonlinear model**	$$\begin{array}{l} \Delta x=2.5mm\\ \Delta y=3.1mm\\ \Delta z=\text{0}\text{.3}mm \end{array}$$	$$\begin{array}{l} \Delta x=3.0mm\\ \Delta y=3.1mm\\ \Delta z=\text{0}\text{.9}mm \end{array}$$	$$\begin{array}{l} \Delta x=3.2mm\\ \Delta y=4.0mm\\ \Delta z=\text{0}\text{.3}mm \end{array}$$	$$\begin{array}{l} \Delta x=2.9mm\\ \Delta y=2.8mm\\ \Delta z=\text{0}\text{.7}mm \end{array}$$	$$\begin{array}{l} \Delta x=2.6mm\\ \Delta y=2.4mm\\ \Delta z=\text{0}\text{.4}mm \end{array}$$	$$\begin{array}{l} \Delta x=3.0mm\\ \Delta y=2.9mm\\ \Delta z=\text{1}\text{.1}mm \end{array}$$

**Mean error of the nonlinear model**	$$\begin{array}{l} \Delta x=0.8mm\\ \Delta y=1.1mm\\ \Delta z=\text{0}\text{.1}mm \end{array}$$	$$\begin{array}{l} \Delta x=1.2mm\\ \Delta y=0.8mm\\ \Delta z=\text{0}\text{.2}mm \end{array}$$	$$\begin{array}{l} \Delta x=1.1mm\\ \Delta y=1.3mm\\ \Delta z=\text{0}\text{.1}mm \end{array}$$	$$\begin{array}{l} \Delta x=0.8mm\\ \Delta y=0.9mm\\ \Delta z=\text{0}\text{.4}mm \end{array}$$	$$\begin{array}{l} \Delta x=0.7mm\\ \Delta y=0.7mm\\ \Delta z=\text{0}\text{.1}mm \end{array}$$	$$\begin{array}{l} \Delta x=0.8mm\\ \Delta y=0.5mm\\ \Delta z=\text{0}\text{.9}mm \end{array}$$

Here, pre-operative and intra-operative MRI studies of six patients undergoing brain tumor surgery are used. The pre- and intra-operative images are registered rigidly, and then pairs of anatomical landmarks are determined by an expert radiologist in the corresponding images. One half of the points are used in the optimization process and the other half are used in the testing process. It should be mentioned that the overall mean of displacement in the x, y, and z direction for the linear mechanical model are 1.2, 1, and 0.3, respectively. These values for the nonlinear model are 0.9, 0.8, and 0.3, respectively. Based on the error of the testing landmarks, the proposed methods are evaluated and compared. Note that the nonlinear model in terms of both the maximum and the mean error in most cases has better results than the linear mechanical model.

**Table 4:** Variations of the estimated parameters of the linear mechanical and nonlinear models.

	Optimized in six cases	Model
**E**	3000 ± 452	**Mechanical**
	
*ν*	0.45 ± 0.03	
	
**Resultant Force**	91 -- 710	

**C_01_**	253 ± 45	**Nonlinear**
	
**C_10_**	253 ± 52	
	
**C_20_**	491 ± 73	
	
**C_02_**	491 ± 73	
	
**g_1_+g_2_**	0.82 ± 0.03	
	
**Resultant Force**	121 -- 852	

These are the results of performing models on the six cases relative to the initial values.

The execution time of the nonlinear model is approximately six times of the linear mechanical model using a personal computer with a 1.86 GHz dual-core CPU and 4 GB RAM. This ratio is an approximation because the execution time depends on the problem complexity, the number of slices, and the mesh resolution that are different for different cases.

This method can be used for estimating the deformation of the brain after opening the skull for brain surgery, and calculates the displacements of the anatomical landmarks on the exposed surface of the brain. By optimizing the model parameters for each patient, the prediction accuracy increases. In addition, devices like neuro-navigators and lasers can be used to determine the coordinates of pre-operative points corresponding to specific intra-operative points. This method does not use intra-operative images. Moreover, by defining a pattern for the force parameter in the proposed models based on specifications like the tumor depth and the exposed surface, approximate parameters of the models can be determined and used in the models to estimate the deformation of the brain without the optimization process.

## Conclusion

We have presented a brain shift compensation method based on linear and nonlinear biomechanical models guided by limited intra-operative data. To this end, we have employed finite element methods for descritizing and solving partial differential equations that describe the brain deformation and optimized their parameters for each case for reducing the inaccuracy due to the variations of the parameters from case to case. Also, we have presented a new procedure for defining the force parameter for the models.

To evaluate the proposed method, we have used simulations as well as real MRI data of the brain. Experimental results have shown that both of the linear mechanical and nonlinear models generate shape deformations similar to the brain deformations.

In their applications to a simulation study, the nonlinear model generated the most accurate displacements and the linear mechanical model generated more accurate displacements than the linear elastic model. In addition, in their applications to the real data, the nonlinear model generated the best matching for the tumor while the linear mechanical model outperformed the linear elastic model. The landmarks near the exposed surface showed superiority of the nonlinear model based on the maximum and mean error of the surface landmarks not used in the optimization process.

From the computation point of view, the linear mechanical model is about 66% slower than the elastic model and six times faster than the nonlinear model. Therefore, depending on the desired levels of speed and accuracy, one of the models can be used. The results of our study confirm that the brain deformation can be reliably estimated using anatomical landmarks on the exposed surface of the brain that can be easily measured by the neuro-navigators used in the operation rooms.

Last but not least, the proposed optimization process eliminates the prediction errors due to the variations of the model parameters from patient to patient. It also confirms the conclusion of [23] that the results of the linear and nonlinear model are not considerably different and thus, considering the execution speed of the two models, the linear mechanical model may be selected for the modeling of the brain deformation.

## Competing interests

The authors declare that they have no competing interests.

## Authors' contributions

HH implemented the algorithms presented in the manuscript, tested the algorithms, and prepared first draft of the manuscript. HS participated in the design and coordination of the study, helped with the testing and evaluation of the proposed methods, and revised the manuscript. RF helped with the implementation and application of the finite element algorithms and evaluation of the simulation results. MG identified the landmarks on the MR images and helped with the evaluation of the clinical results. All authors read and approved the final manuscript.
